# CCAAT/Enhancer-Binding Protein γ Is a Critical Regulator of IL-1β-Induced IL-6 Production in Alveolar Epithelial Cells

**DOI:** 10.1371/journal.pone.0035492

**Published:** 2012-04-27

**Authors:** Chunguang Yan, Ximo Wang, Jay Cao, Min Wu, Hongwei Gao

**Affiliations:** 1 Center for Experimental Therapeutics and Reperfusion Injury, Department of Anesthesiology, Perioperative and Pain Medicine, Brigham and Women's Hospital, Harvard Medical School, Boston, Massachusetts, United States of America; 2 Department of Biochemistry and Molecular Biology, University of North Dakota, Grand Forks, North Dakota, United States of America; 3 Department of Surgery, Tianjin Union Medical Center, Nankai University Affiliated Hospital, Tianjin, China; 4 Agricultural Research Service, Grand Forks Human Nutrition Research Center, United States Department of Agriculture, Grand Forks, North Dakota, United States of America; National Jewish Health, United States of America

## Abstract

CCAAT/enhancer binding protein γ (C/EBPγ) is a member of the C/EBP family of transcription factors, which lacks known activation domains. C/EBPγ was originally described as an inhibitor of C/EBP transactivation potential. However, previous study demonstrates that C/EBPγ augments the C/EBPβ stimulatory activity in lipopolysaccharide induction of IL-6 promoter in a B lymphoblast cell line. These data indicate a complexing functional role for C/EBPγ in regulating gene expression. Furthermore, the expression and function of C/EBPγ during inflammation are still largely unknown. In this study, we demonstrate that C/EBPγ activation was induced by IL-1β treatment in lung epithelial cells. Importantly, we demonstrate for the first time that C/EBPγ plays a critical role in regulating IL-1β-induced IL-6 expression in both mouse primary alveolar type II epithelial cells and a lung epithelial cell line, MLE12. We further provide the evidence that C/EBPγ inhibits IL-6 expression by inhibiting C/EBPβ but not NF-κB stimulatory activity in MLE12 cells. These findings suggest that C/EBPγ is a key transcription factor that regulates the IL-6 expression in alveolar epithelial cells, and may play an important regulatory role in lung inflammatory responses.

## Introduction

As one of the major cell types comprising alveolar epithelial tissue, the alveolar type II epithelial cells play an important role in maintaining alveolar integrity by forming the key alveolar barrier, repairing damaged type I cells, and being the source of alveolar surfactant [1], [2], [3]. Increasing studies also suggest a critical role for alveolar type II epithelial cells in regulating local lung inflammatory response. For example, our previous study and others have suggested that alveolar type II epithelial cells may play special roles in counteracting microbes by releasing cytokines and chemokines that recruit both dendritic cells and alveolar macrophages to the site of infection [4], [5], . However, the potential role of alveolar type II epithelial cells in lung innate immunity and the molecular mechanisms whereby the expressions of inflammatory mediators are regulated in alveolar type II epithelial cells remain largely unknown. IL-1β is one of the most biologically active cytokines in edema fluid and bronchoalveolar lavage (BAL) fluid from patients at an early stage of acute respiratory distress syndrome (ARDS). Moreover, IL-1β has been shown to affect the function of the lung epithelial barrier. IL-1β is known to modulate the activity of many transcription factors including NF-κB and C/EBPs. However, the role of C/EBPs in IL-1β-mediated inflammatory responses in alveolar type II epithelial cells remains unknown. The goal of the current study was to investigate the role of C/EBPγ in IL-1β-stimulated IL-6 production from alveolar type II epithelial cells.

C/EBPα, -β, -δ, -ε, -γ, and -ζ comprise a family of basic region-leucine zipper (bZIP) transcription factors that dimerize through a leucine zipper and bind to DNA through an adjacent basic region. All C/EBP members can form homo- and hetero-dimers with other family members. C/EBPs can activate transcription from promoters that contain a consensus binding site: 5′-T(T/G)NNGNAA(T/G)-3′ [8]. Among them, C/EBPβ and -δ appear to be effectors in the induction of genes responsive to LPS, IL-1β or IL-6 stimulation, and have been implicated in the regulation of inflammatory mediators as well as other gene products associated with the activation of macrophages and the acute phase inflammatory response [9] [10], [11]. C/EBPγ is a ubiquitously expressed member of the C/EBP family of transcription factors that has been shown to be an inhibitor of C/EBP transcriptional activators. Different from C/EBPβ and -δ, C/EBPγ was proposed to act as a buffer against C/EBP-mediated activation because of the fact that C/EBPγ lacks known activation domains [12]. C/EBPγ-deficient mice showed a high mortality rate within 48 h after birth [13]. Although C/EBPγ chimeras showed normal T and B cell development, the cytolytic functions of their splenic natural killer cells after stimulation with cytokines such as IL-12, IL-18 and IL-2 were significantly reduced [13]. However, the role of C/EBPγ in inflammation remains largely unknown. In the current study, we demonstrate that C/EBPγ expression is induced by IL-1β in lung epithelial cells, and apparently contributes to the inhibition of IL-1β-mediated IL-6 production. Furthermore, we show that C/EBPγ inhibits IL-6 expression by inhibiting C/EBPβ stimulatory activity. In sharp contrast, NF-κB activity is not impaired by C/EBPγ. The data suggest that C/EBPγ may play an important regulatory role in lung inflammatory responses.

## Results

### C/EBPγ suppresses IL-1β-induced IL-6 production in MLE12 cells

**Figure 1:**
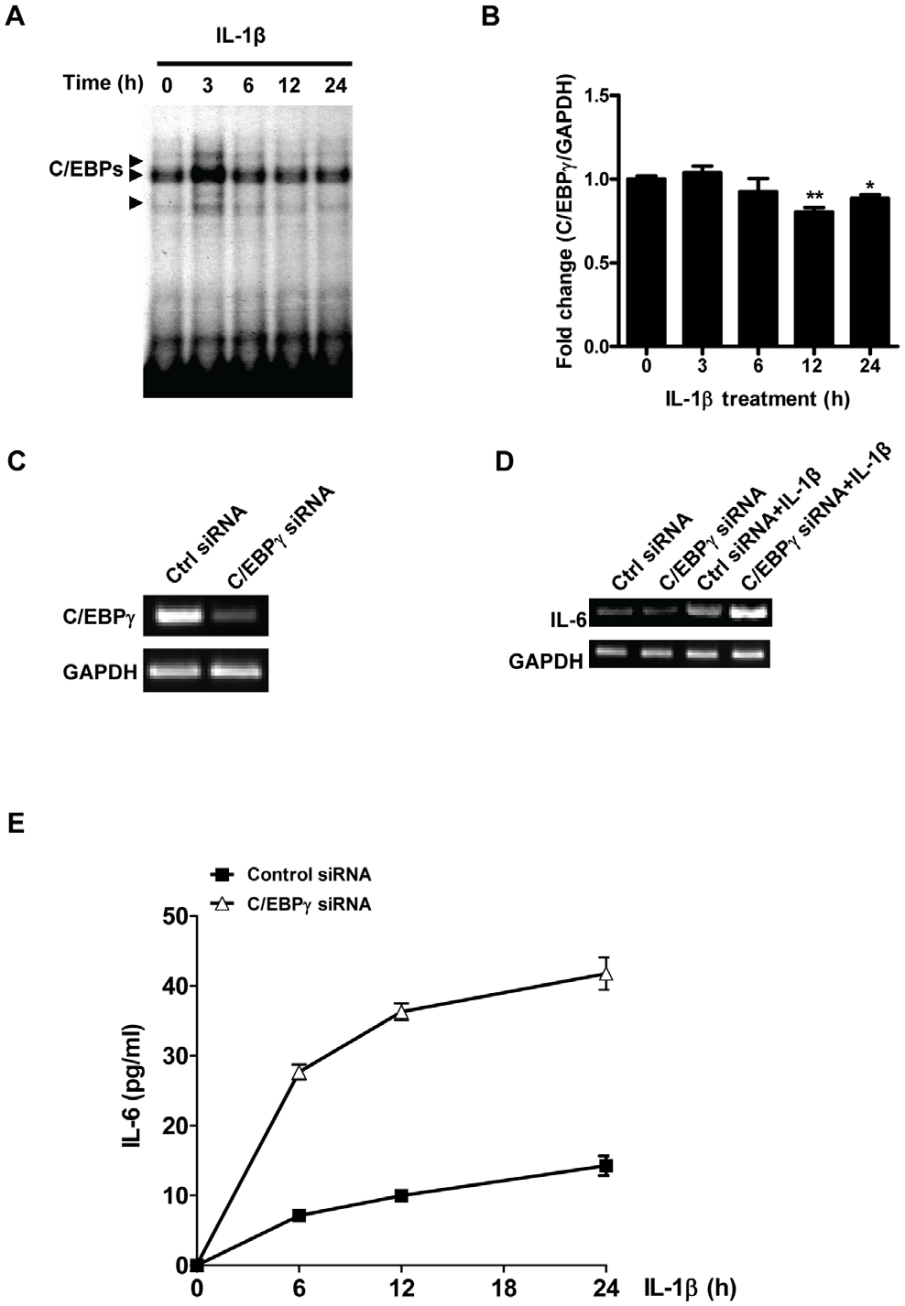
Knockdown of C/EBPγ enhances IL-6 production in MLE12 cells. MLE12 cells were treated with 20 ng/ml IL-1β for different time periods. A, the nuclear proteins were harvested. Activation of C/EBPs was detected by EMSA. B, total cellular RNA was extracted for Real time-PCR with primers for C/EBPγ and GAPDH, respectively. C, the cells were transiently transfected with control siRNA, and C/EBPγ-specific siRNA, respectively. 24 h after transfection, RNAs were isolated and RT-PCR was performed by using primers for C/EBPγ, and GAPDH, respectively. D and E, MLE12 cells were transiently transfected with control siRNA or C/EBPγ-specific siRNA. 24 h later, cells were stimulated by 20 ng/ml IL-1β for indicated time periods. D, RNAs were harvested for RT-PCR using primers for IL-6, and GAPDH, respectively. E, the supernatants were harvested for ELISA. Data were means ± S. E. M., N = 8.

Little is known about the expression and function of C/EBPγ during inflammation. We evaluated the time course of C/EBP activation in lung epithelial cells by EMSA, using nuclear extracts from MLE12, a lung epithelial cell line, obtained at various time points after IL-1β treatment. As shown in Fig. 1A, at time 0, low levels of constitutive C/EBPs (mainly C/EBPβ and C/EBPγ) were observed. C/EBP activation became stronger by 3 h, then decreased to control levels (Fig. 1A). To determine if increased oligonucleotide binding to C/EBPγ was caused by increased transcriptional expression of C/EBPγ, we conducted Real Time PCR experiments. As shown in Fig. 1B, constitutive levels of C/EBPγ mRNA expression in MLE12 cells were observed at 0, 3, and 6 h after IL-1β treatment, with modest decreases at 12 and 24 h. These data suggest that the C/EBPγ DNA binding was mainly regulated at post-transcriptional level. C/EBPβ has been shown to participate in IL-1β signaling such as mediating IL-6 production [14]. However, the role of C/EBPγ in IL-1β signaling is unclear. We therefore determined whether C/EBPγ expression in MLE12 cells has any effect on IL-1β-induced IL-6 production. MLE 12 cells were transfected with control siRNA or C/EBPγ siRNA before IL-1β challenge. We demonstrated that C/EBPγ siRNA could effectively suppress the endogenous C/EBPγ expression in MLE12 cells (Fig. 1C). We found that knockdown of C/EBPγ significantly increased IL-1β-stimulated IL-6 expression at mRNA level (Fig. 1D). Moreover, we show that IL-6 production at protein level was increasingly elevated in a time-dependent manner when the MLE12 cells were stimulated with IL-1β (Fig. 1E). Importantly, knockdown of C/EBPγ in MLE12 cells led to a significant increase of IL-1β-stimulated IL-6 secretion (more than 300% increase) at all time points when compared with control group (Fig. 1E), suggesting a negative regulatory role of C/EBPγ in IL-1β-induced IL-6 expression.

**Figure 2:**
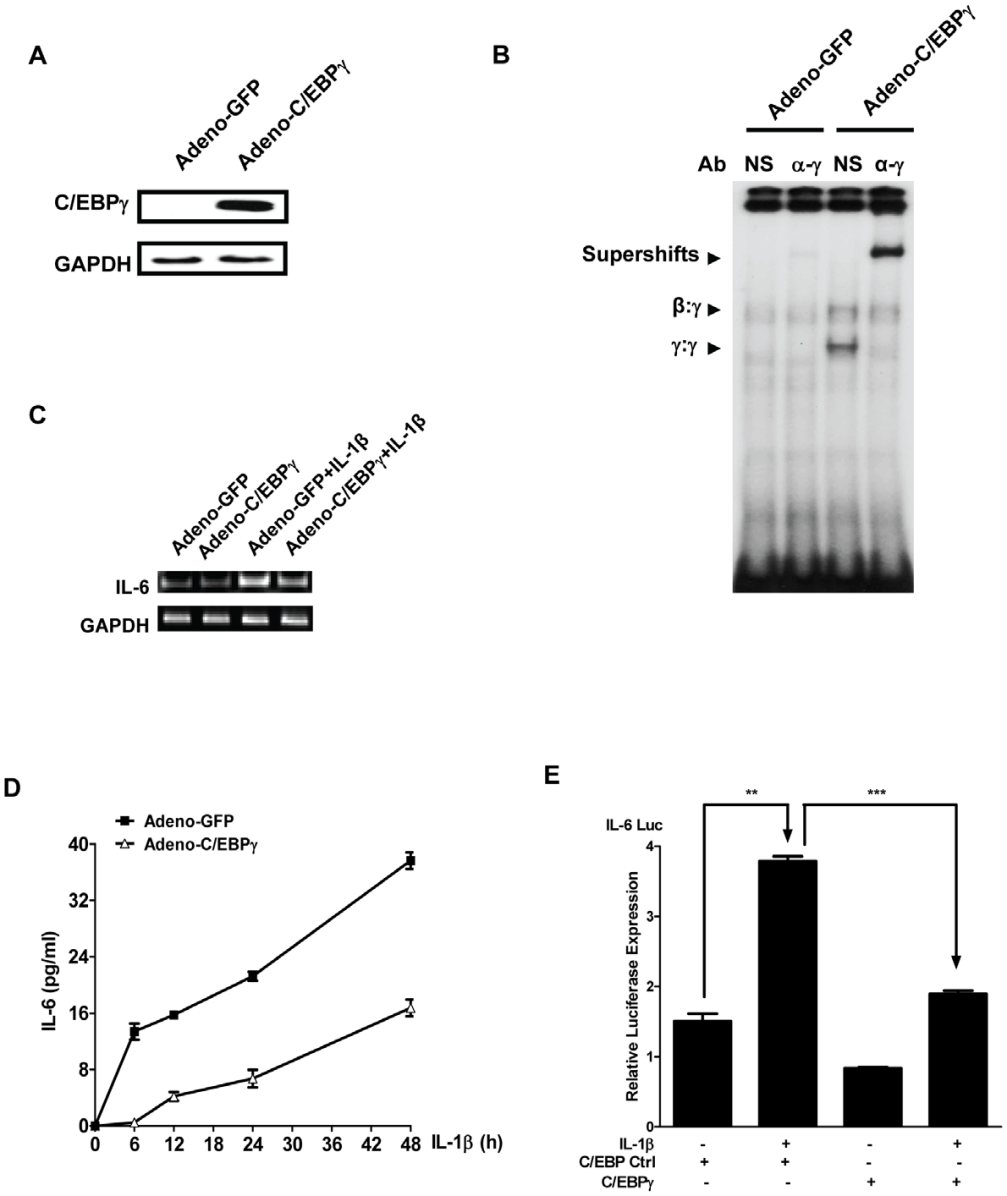
Ectopic expression of C/EBPγ inhibits IL-1β-mediated IL-6 expression in MLE12 cells. A, MLE12 cells were infected with control adenovirus (Adeno-GFP) or C/EBPγ expressing adenovirus (Adeno-C/EBPγ) at 20 MOI. 24 h after infection, total protein were harvested and subjected to Western blot using antibodies against C/EBPγ and GAPDH, respectively. B, MLE12 cells were infected with Adeno-GFP or Adeno-C/EBPγ at 20 MOI. 24 h later, the nuclear extracts were subjected for EMSA. NS and α-γ represent normal rabbit IgG, and anti-C/EBPγ antibody, respectively. Arrows indicated C/EBP binding species and supershift bands. C and D, MLE12 cells were infected with Adeno-GFP or Adeno-C/EBPγ at 20 MOI. 24 h later, the cells were stimulated with or without 20 ng/ml IL-1β for indicated time periods. C, RNAs were isolated and RT-PCR was conducted using primers for IL-6 and GAPDH, respectively. D, the supernatants were harvested for ELISA. Data were expressed as means ± S. E. M., N = 8. E, MLE12 cells were transfected with indicated plasmids. 24 h later, the cells were treated with or without 20 ng/ml IL-1β. Cell lysates were subjeceted for luciferase activity measurement. Luminometer values were normalized for expression from a co-transfected thymidine kinase reporter gene. The data were expressed as means of three experiments ± S. E. M. ** and *** indicated statistically significant difference-*p*<0.01 and *p*<0.001, respectively.

To further determine the C/EBPγ regulation of IL-6 expression, we infected MLE12 cells with adenovirus that could induce C/EBPγ expression (Adeno-C/EBPγ). As shown in Fig. 2A, cells infected with Adeno-C/EBPγ exhibited high level of C/EBPγ protein expression. We further demonstrated that the exogenously expressed C/EBPγ can bind to C/EBP binding site in the IL-6 promoter by EMSA (Fig. 2B). We next showed that C/EBPγ expression significantly suppressed IL-1β-induced IL-6 expression at both mRNA and protein levels (Fig. 2C and D). To further determine the ability of C/EBPγ to suppress IL-1β-induced IL-6 expression, MLE12 cells were transfected with an IL-6 promoter-luciferase construct together with C/EBPγ plasmid or control vector in the presence or absence of IL-1β. As shown in Fig. 2E, IL-1β stimulation induced IL-6 promoter-driven luciferase expression by over 2.3-fold. However, C/EBPγ over-expression led to an over 50% reduction in the luciferase expression (Fig. 2E).

### C/EBPγ suppresses IL-1β-induced IL-6 expression in primary alveolar type II epithelial cells

**Figure 3:**
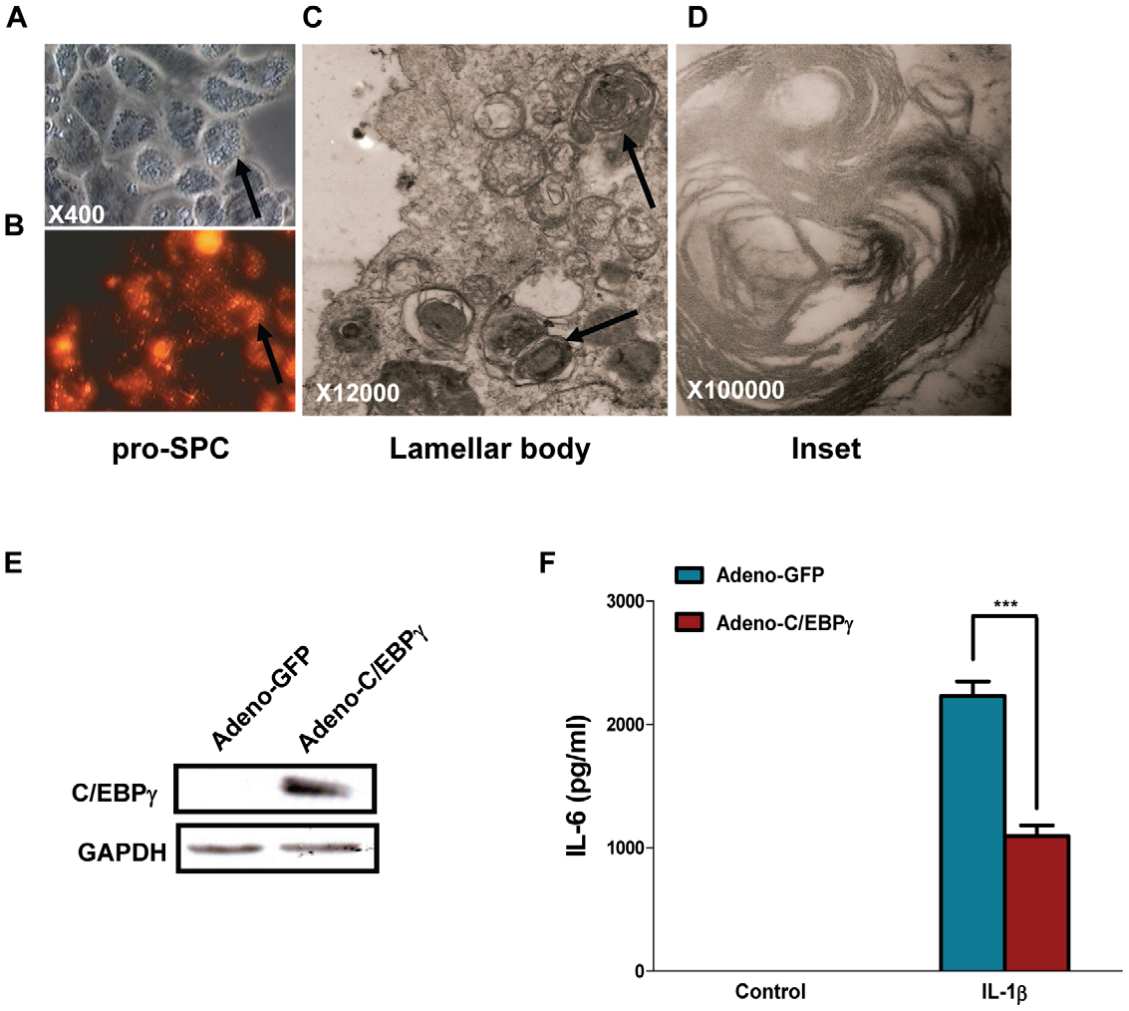
Over-expression of C/EBPγ suppresses IL-1β-induced IL-6 production in primary cultured alveolar type II epithelial cells. A–D, alveolar type II epithelial cells were isolated from C57BL6 mouse, and identified by pro-SP-C immunostaining (A, B), as well as lamellar body transmission electron microscopy. A, B, and C indicate DIC, fluorescence, and transmission electron microscopy, respectively; and D indicates enlarged lamellar body. Arrows in A and B indicate staining for pro-SP-C and in C indicate lamellar body. E, alveolar type II epithelial cells were infected with Adeno-GFP or Adeno-C/EBPγ at a MOI of 50. 24 h after infection, the total protein were harvested and subjected for Western blot by using antibodies against C/EBPγ and GAPDH, respectively. F, alveolar type II epithelial cells were infected with Adeno-GFP or Adeno-C/EBPγ at a MOI of 50. 24 h later, the cells were cultured with or without 50 ng/ml IL-1β for another 24 h. The supernatants were subjected for ELISA. Data were expressed as means ± S. E. M., N = 10.

To further confirm the inhibitory role of C/EBPγ in IL-6 expression observed in the MLE12 cells, we isolated primary alveolar type II epithelial cells from mouse lung. As shown in Fig. 3A and B, expression of the surfactant protein C was confirmed using fluorescent staining with a pro-SP-C monoclonal antibody. The successful isolation of primary alveolar type II epithelial cells was verified using TEM assay that shows the lamellar bodies of characteristic features (Fig. 3 C and D). Primary alveolar type II epithelial cells were infected with Adeno-GFP and Adeno-C/EBPγ, respectively. Our preliminary study shows that adenoviral transfection efficiency is about 40% (data not shown). Consistent with the results obtained from MLE12 cells, we found that over-expression of C/EBPγ significantly inhibited IL-6 secretion after IL-1β stimulation (Fig. 3F). Taken together, these results support the inhibitory role of C/EBPγ on IL-1β-induced IL-6 production in alveolar type II epithelial cells.

### IL-1β induces the activation of both C/EBPβ/γ and NF-κB in MLE12 cells

**Figure 4:**
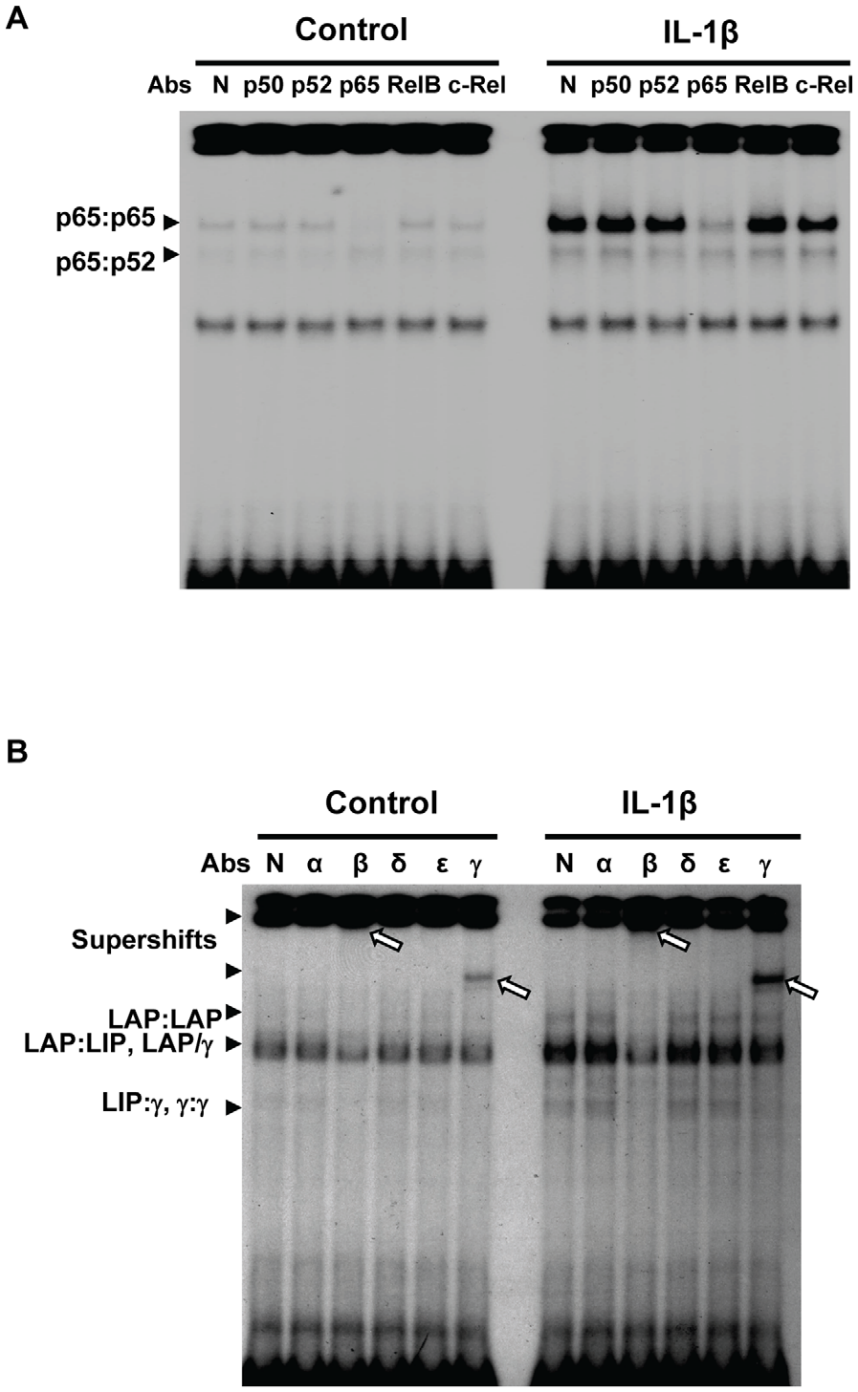
C/EBPβ and NF-κB are activated by IL-1β treatment in MLE12 cells. MLE12 cells were stimulated with 20 ng/ml IL-1β for 3 h. The nuclear proteins were harvested. Activation of NF-κB (A) and C/EBP (B) were detected by EMSA. N, α, β, δ, ε, and γ represent normal rabbit IgG, anti-C/EBPα antibody, anti-C/EBPβ antibody, anti-C/EBPδ antibody, anti-C/EBPε antibody, and anti-C/EBPγ antibody, respectively. Arrows indicated C/EBPs, NF-κB binding species and supershift bands.

Previous studies including ours show that C/EBPβ and NF-κB synergistically activate the IL-6 expression in various immune cells. Thus, we examined the activation of C/EBPs and NF-κB in IL-1β-treated MLE12 cells. As shown in Fig. 4A, IL-1β induces strong NF-κB DNA-binding activity (mainly p65) in MLE12 cells. Furthermore, IL-1β treatment also led to the induction of C/EBP DNA-binding activity in the MLE12 cells (Fig. 4B). The C/EBPβ gene can produce several N-terminally truncated isoforms including Liver-enriched activator protein (LAP) and liver-enriched inhibitory protein (LIP) [15]. LAP is a transcriptional activator in many systems, whereas the function of LIP is controversial. Using supershift assay, we found that C/EBP DNA binding species contained both C/EBPβ and C/EBPγ, in IL-1β-treated and untreated cells (Fig. 4B). Moreover, IL-1β induced the DNA-binding activity of C/EBPγ (mainly in the forms of C/EBPγ:γ homodimers and C/EBPγ:LAP/LIP heterodimers).

### C/EBPβ and p65 are indispensable for IL-6 expression in MLE12 cells

**Figure 5:**
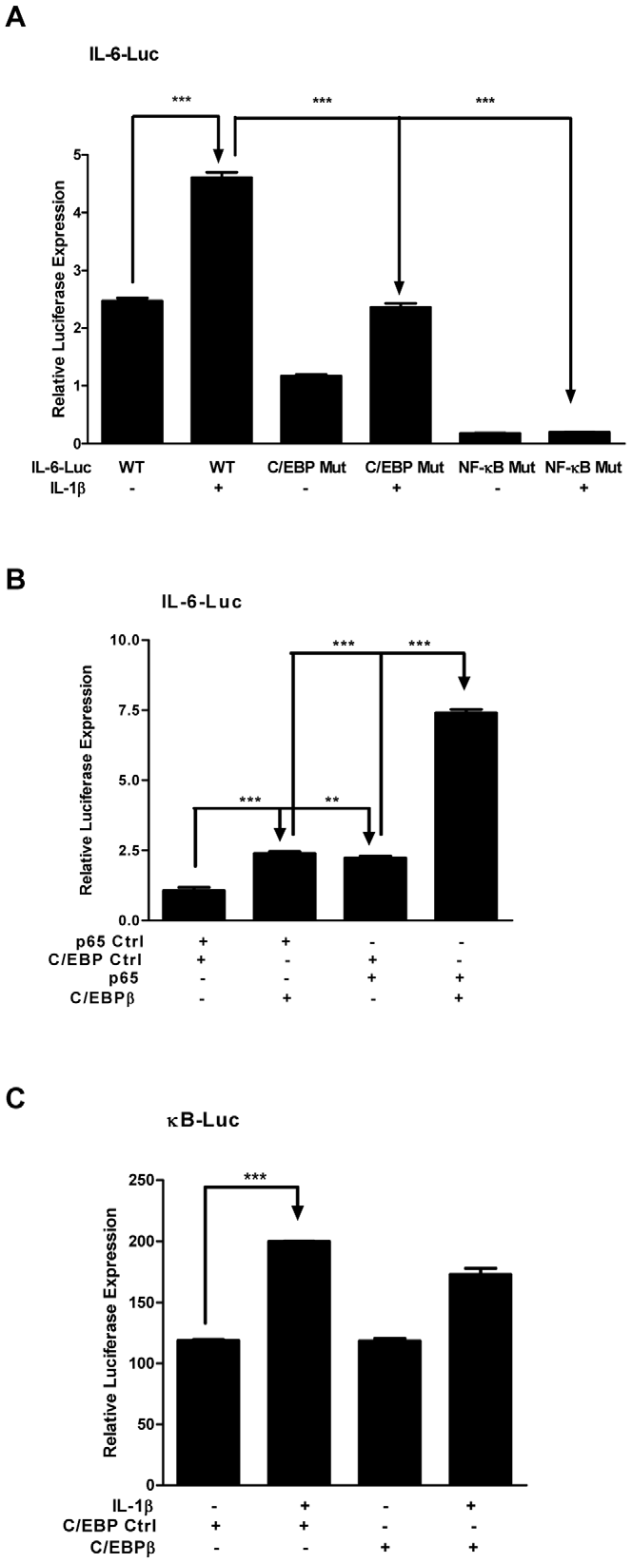
C/EBPβ and NF-κB p65 are involved in IL-1β-induced IL-6 expression. A, B and C. MLE12 cells were transfected with indicated plasmids. 24 h later, the cells were treated with or without 20 ng/ml IL-1β. The cell lysates were subjected for luciferase activity measurement. Luminometer values were normalized for expression from a co-transfected thymidine kinase reporter gene. The data were expressed as means of three experiments ± S. E. M. ** and *** indicated statistically significant difference-*p*<0.01, and *p*<0.001, respectively.

To determine if interaction of both NF-κB and C/EBPβ with the IL-6 promoter region was required for the IL-1β-induced IL-6 expression in MLE12 cells, we transfected MLE12 cells with an IL-6 promoter-luciferase construct or an L-6 promoter-luciferase construct harboring a mutant in either the NF-κB binding site or the C/EBP binding site. As shown in Fig. 5A, a mutation in either the NF-κB binding site or the C/EBP binding site led to a significant decrease of IL-6 promoter-luciferase activity following IL-1β stimulation compared with non-mutated IL-6 promoter-luciferase. We further determined the ability of NF-κB and C/EBPβ to synergistically induce the IL-6 promoter-luciferase activity in MLE12 cells. As shown in Fig. 5B, transient transfection with p65 or C/EBPβ expression vector caused an over 2-fold increase of luciferase activity when compared with the control vector. Concurrent forced expression of p65 and C/EBPβ led to a 3-fold increase of promoter activity compared with p65 or C/EBPβ alone (Fig. 5B). We next examined if the synergistic effect was due to the increased p65 DNA binding activity induced by C/EBPβ expression. As shown in Fig. 5C, C/EBPβ expression could marginally affect IL-1β-stimulated NF-κB DNA binding activity. Therefore, interaction between p65 and C/EBPβ might be involved in their synergistic activation of IL-6 promoter activity induced by IL-1β.

**Figure 6:**
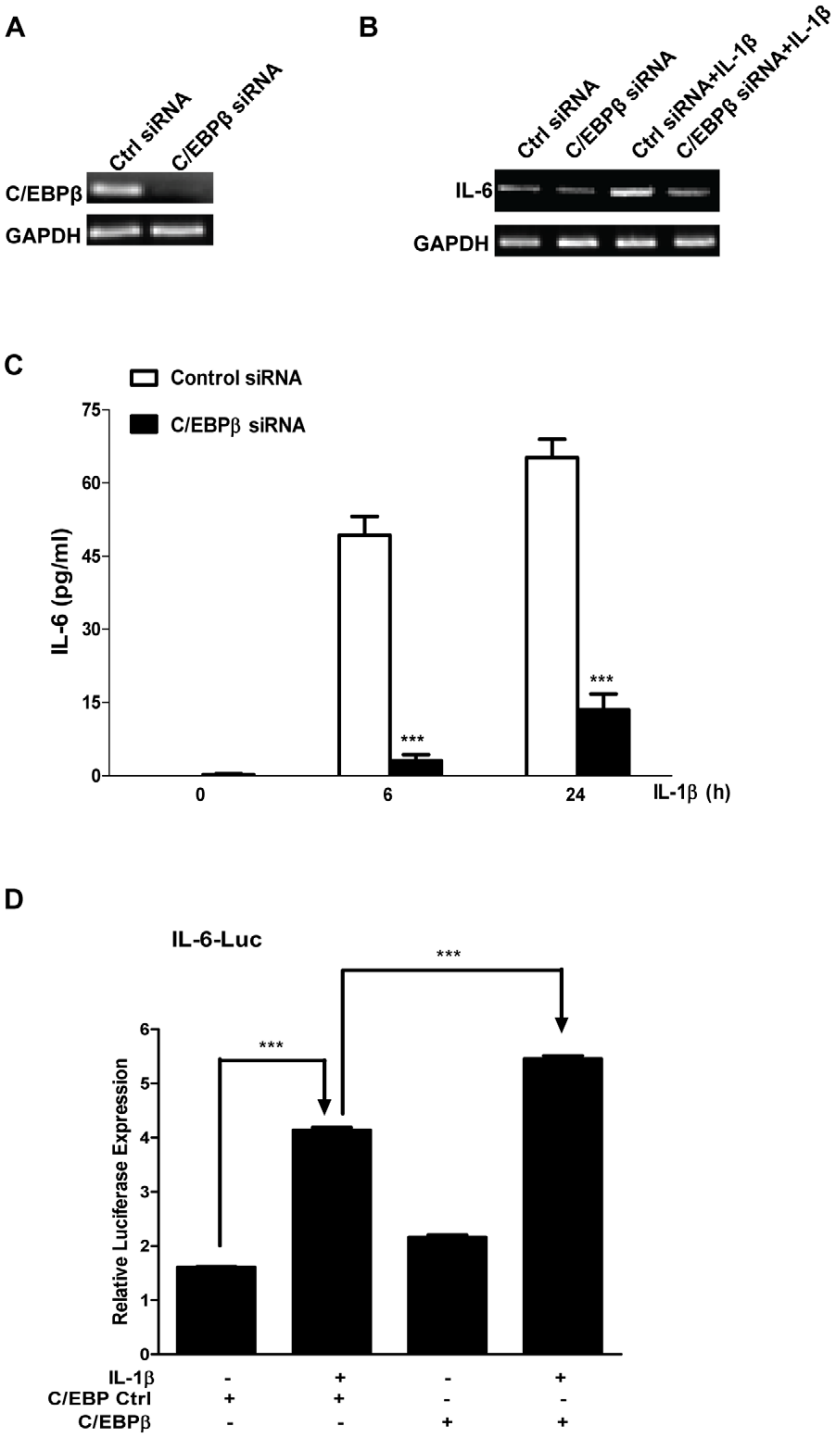
Knockdown of C/EBPβ by siRNA inhibits IL-1β-induced IL-6 production in MLE12 Cells. A, MLE12 cells were transfected with 40 nM control siRNA or C/EBPβ-specific siRNA. 24 h later, RNAs were isolated and RT-PCR was performed by using primers for C/EBPβ and GAPDH, respectively. B and C, MLE12 cells were transfected with 40 nM control siRNA or C/EBPβ si RNA. 24 h later, the cells were incubated with or without 20 ng/ml IL-1β for different time periods. B, RNAs were isolated and RT-PCR was performed by using primers for IL-6 and GAPDH, respectively. C, Supernatants were harvested for ELISA. The data were expressed as means± S. E. M., N = 8. D, MLE12 cells were transfected with indicated plasmids. 24 h later, the cells were treated with 20 ng/ml IL-1β for 6 h. Cell lysates were subjected for luciferase activity measurement. Luminometer values were normalized for expression from a co-transfected thymidine kinase reporter gene. The data were expressed as means of three experiments ± S. E. M.

To further determine the role of C/EBPβ in IL-1β-induced IL-6 production, we transfected MLE12 cells with control siRNA or siRNA specific for C/EBPβ. As shown in Fig. 6A, C/EBPβ siRNA almost completely abrogated C/EBPβ expression compared with control siRNA in MLE12 cells. Furthermore, knockdown of C/EBPβ expression significantly decreased IL-1β-induced IL-6 expression at both mRNA and protein levels (Fig. 6B and C). We further examined the role of C/EBβ in IL-1β-induced IL-6 expression in transfection study using IL-6 promoter-luciferase assay. Consistent with the results from RT-PCR and ELISAs (Fig. 6B and C), IL-1β stimulation alone (without transfection of a C/EBPβ expression vector) induced a 2.5-fold increase of luciferase activity compared with control group (Fig. 6D). Moreover, IL-1β treatment of C/EBPβ transfectants led to a 25% increase of luciferase activity than the IL-1β stimulation alone (p<0.001).

### C/EBPγ suppresses IL-1β-induced IL-6 expression by inhibiting C/EBPβ activity but not NF-κB activity

**Figure 7:**
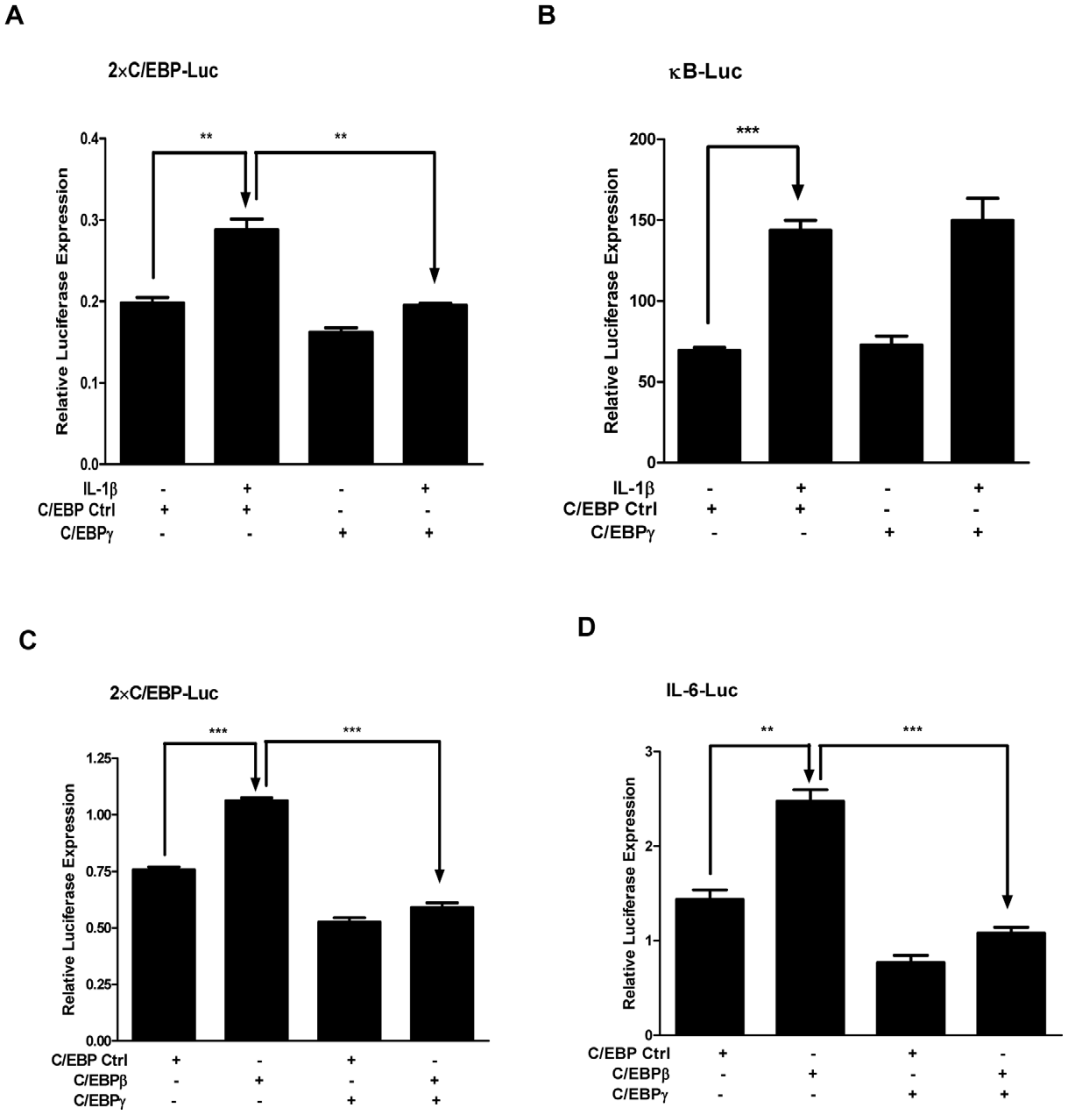
C/EBPγ suppresses IL-1β-induced IL-6 expression by inhibiting C/EBPβ, not NF-κB activation in MLE 12 cells. A–D, MLE12 cells were transfected with indicated plasmids. 24 h later, cells were treated with or without 20 ng/ml IL-1β. The cell lysates were subjected for luciferase activity measurement. Luminometer values were normalized for expression from a co-transfected thymidine kinase reporter gene. The data were expressed as means of three experiments ± S. E. M. ** and *** indicated statistically significant difference-*p*<0.01 and *p*<0.001, respectively.

We reason that C/EBPγ suppresses the IL-6 expression through inhibiting stimulatory C/EBP acitivity. MLE 12 cells were transfected with 2×C/EBP-Luc, a C/EBP-dependent promoter-reporter containing two copies of a C/EBP binding site, together with C/EBPγ expressing plasmid or control plasmid. As shown in Fig. 7A, IL-1β stimulation led to a significant increase of 2×C/EBP-Luc expression, and over-expression of C/EBPγ resulted in a reduction of luciferase activity to the basal level. In sharp contrast, although there is a more than 2-fold IL-1β induction of κB-Luciferase expression, this activity was not affected by C/EBPγ expression (Fig. 7B). We further show that C/EBPγ over-expression caused a significant decrease of the 2×C/EBP-Luc expression induced by C/EBPβ over-expression (Fig. 7C). To determine if decreased C/EBPβ binding by C/EBPγ could lead to the reduced IL-6 expression, MLE 12 cells were transfected with C/EBPβ plasmid in the presence or absence of C/EBPγ plasmid. As shown in Fig. 7D, C/EBPβ itself caused a 1.7-fold increase of IL-6-Luc expression (P<0.01), while over-expression of C/EBPγ led to a significant decrease of the luciferase expression. Together, these data suggest that C/EBPγ inhibits IL-1β-induced IL-6 expression by suppressing C/EBPβ activity.

## Discussion

Previous study shows that C/EBPγ dramatically augments the activity of C/EBPβ in LPS induction of the IL-6 and IL-8 promoters in a B lymphoblast cell line [16]. In another study, Kaisho et al show that the ability of C/EBPγ (-/-) chimera splenocytes to produce interferon γ in response to IL-12 and/or IL-18 was markedly impaired [13]. To our knowledge, these are the only two reports indicating a possible role of C/EBPγ in regulating the expression of inflammatory mediators. In this study, we show that C/EBPγ expression is induced by IL-1β in alveolar type II epithelial cells. We further show that C/EBPγ is a critical regulator of IL-1β-mediated IL-6 production.

Although alveolar type II epithelial cells only cover about 5% of the alveolar surface area, there is increasing evidence that they play a significant role in lung inflammatory diseases. IL-1β is a critical inducer of immune and inflammatory responses by mediating activation of alveolar type II epithelial cells, leading to pro-inflammatory cytokine production such as IL-6 [17]. IL-6, which is a pleiotropic cytokine produced by a variety of cell populations such as alveolar macrophages and alveolar type II cells, plays an important role in both acute and chronic lung injury [18], [19]. IL-6 expression is mainly regulated at transcriptional level, which is controlled by a variety of transcription factors binding to the *cis*-acting elements of the IL-6 promoter region, such as NF-κB and C/EBPβ/δ. For example, C/EBPβ and -δ have both been shown to activate a reporter gene controlled by the IL-6 promoter in transient expression assays [8], [20]. Furthermore, the stable expression of C/EBPβ in a murine B lymphoblast cell line is sufficient to confer LPS inducibility of IL-6 expression [21]. Importantly, NF-κB and C/EBPβ synergistically activate the IL-6 promoter, and consistent with this, direct interaction between the C/EBPβ bZIP and the NF-κB Rel homology domain has been observed [22], as well as cooperative binding of the two factors [22]. In addition, the NF-κB site of the IL-6 promoter is required for the activity of the C/EBPβ bZIP in the absence of amino-terminal motifs [23]. All of these studies suggested a mechanism for IL-6 activation whose essential feature is the requirement for the bZIP region of C/EPBβ to synergize with NF-κB, although this remains to be further investigated. In addition, it has been recently shown that IL-1β-induced IL-6 production in alveolar type II cells is associated with the activation of both IL-1 receptor-associated kinase-4 and phosphatidylinositol 3-kinase [24]. However, in alveolar type II cells, molecular mechanisms involved in IL-1β-induced IL-6 production remains largely unknown. In the current study, we find that the binding activity of both NF-κB and C/EBPβ to their regulatory elements in the IL-6 promoter is significantly elevated by IL-1β stimulation in alveolar epithelial cells. Our data further indicate that both C/EBPβ and p65 are indispensable for IL-1β-induced IL-6 expression, which is consistent with the observation in other cell types.

Our finding that C/EBPγ can regulate IL-1β-induced IL-6 production in alveolar type II epithelial cells is interesting. C/EBPγ has been considered as an inhibitor of other C/EBP family members. For example, C/EBPγ inhibits C/EBPβ-mediated HIV-1 long terminal repeat-driven transcription in human brain cells [25]. In addition, C/EBPγ represses C/EBPβ-mediated induction of alcohol dehydrogenase expression in the rat livers [16]. These results are consistent with the fact that C/EBPγ lacks known activation domains and is essentially a C/EBP bZIP domain [12]. In contrast, C/EBPγ can also act as a transcription activator. For example, C/EBPγ has been shown to be a positive regulator of IFN-γ expression in splenocytes and NK cells [13], and gamma-globin expression in fetal liver [26]. Furthermore, previous studies demonstrated that augmentation of C/EBPβ activity on the IL-6 and IL-8 promoters by C/EBPγ required formation of a heterodimeric leucine zipper and co-expression of NF-κB [16]. Interestingly, C/EBPγ inhibits C/EBPβ- and C/EBPδ-mediated transactivation of a reporter gene in fibroblasts in a leucine zipper-dependent manner, but it has no suppressive function in HepG2 hepatoma cells [27]. These findings together indicate that C/EBPγ has complex effects on gene transcription. Our current finding that C/EBPγ suppresses IL-1β-induced IL-6 production in alveolar type II epithelial cells further suggests that its function may be cell specific.

The exact molecular mechanism whereby C/EBPγ regulates gene expression is not clear. In IL-1β-stimulated alveolar epithelial cells, C/EBPγ exists as both homodimers (C/EBPγ:γ) and heterodimers (C/EBPγ:β) (Fig. 4B). It is possible that the lack of one transactivation domain in C/EBPγ:β heterodimers may contribute to the inhibitory effect of C/EBPγ. On the other hand, in C/EBPγ-overexpressed cells, the major C/EBPγ binding species is C/EBPγ:γ homodimers (Fig. 2B), suggesting that C/EBPγ:γ homodimers may compete with the stimulatory C/EBP β:β (LAP:LAP or LAP:LIP) to bind to IL-6 promoter region. In addition, we observed an increased C/EBPγ:β heterodimers (LAP:γ) binding to IL-6 promoter in C/EBPγ-overexpressed cells (Fig. 2B and data not shown). This suggests that there is a free C/EBPβ pool in the nucleus. However, whether C/EBPγ is a preferential dimerization partner for C/EBPβ or C/EBPγ:β heterodimers have a higher affinity than C/EBPβ:β in lung epithelial cells remains an open question. Interestingly, C/EBPγ does not seem to affect NF-κB DNA binding (Fig. 7B and data not shown), suggesting that C/EBPγ has no effect on the synergistic activity between NF-κB and C/EBPβ in IL-6 promoter in alveolar epithelial cells (Fig. 5B).

**Figure 8:**
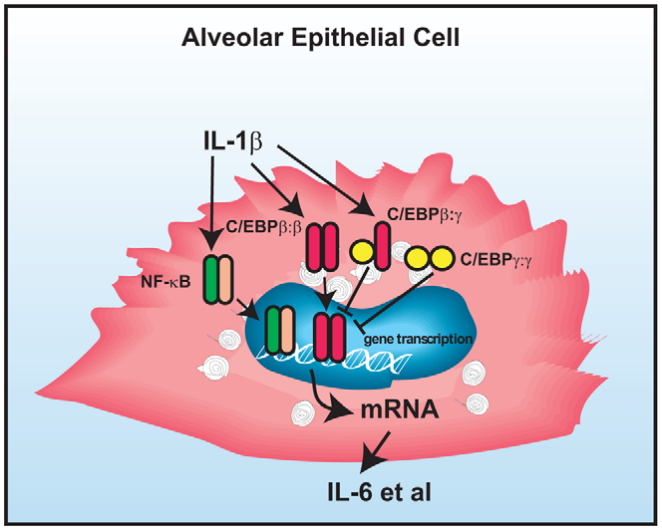
Diagram delineates a role for C/EBPγ in the AECII inflammatory responses. Inflammatory stimuli in the lung such as IL-1β trigger activation of AECII. AECII activation is characterized by increased nuclear translocation, DNA binding of transcription factors such as NF-κB and C/EBPβ, and subsequent production of the pro-inflammatory mediators such as IL-6. This inflammatory response is endogenously regulated by the dominant-negative members of the same family of transcription factors such as C/EBPγ by forming homodimmers themselves and heterodimmers with C/EBPβ to compete with C/EBPβ:β dimmers to bind to promoter regions.

Taken together, we identified a previously unrecognized role for C/EBPγ in inflammation in alveolar epithelial cells. Not surprisingly, many transcription factors such as NF-κB and C/EBPβ are activated in the acute lung inflammatory reaction. However, our current study suggests that the acute inflammatory response in the lung can also be counter-regulated by other transcription factors such as C/EBPγ (Figure 8). Understanding the underlying roles and mechanisms whereby C/EBPγ regulates the network of inflammatory system in the lung may be a crucial step for the development of new therapeutic targets for treatment of lung inflammatory diseases.

## Materials and Methods

### Cells and Reagents

Murine lung epithelial cells (MLE12) and HEK293 cells were obtained from American Type Culture Collection (CRL-2110™, Manassas, VA), and cultured in DMEM/F-12 (Fisher Scientific, Pittsburgh, PA) supplemented with 5% fetal calf serum (FCS, Invitrogen, Carlsbad, CA). Cells were maintained in a humidified incubator at 37°C with 5% CO_2_. Recombinant mouse IL-1β (401-ML) was purchased from R&D Systems, Minneapolis, MN.

### Expression Vectors and Promoter Reporters

Full-length mouse C/EBPγ cDNA was amplified from total RNA of the mouse lung using reverse transcription-PCR (Invitrogen, Carlsbad, CA), sequenced, and inserted into pcDNA3.1(+) (Invitrogen, Carlsbad, CA). Recombinant adenovirus containing mouse C/EBPγ (Adeno-C/EBPγ) was constructed by using BD Adeno-X™ Expression System 1 (BD Biosciences, Palo Alto, CA). To generate the virus, Adeno-C/EBPγ was digested with PacI and transfected to HEK-293 cells according to the manufacturer's instructions. Recombinant adenoviruses were purified by BD Adeno-X virus purification kit (BD Biosciences, Palo Alto, CA), and stored in aliquots at −80°C. The viral stocks were tittered using Adeno-X Rapid Titer Kit (D Biosciences, Palo Alto, CA). The mouse IL-6 promoter-reporter (−250 to +1), IL-6 promoter-reporter containing a mutated NF-κB binding site, as well as the C/EBPβ and NF-κB p65 expression plasmids were kindly provided by Richard C. Schwartz (Michigan State University). Mouse IL-6 promoter-reporter containing a mutated C/EBP binding site (−161 to −147) was kindly provided by Gail A. Bishop (University of Iowa). NF-κB promoter reporter was obtained from Promega. C/EBP promoter reporter, 2×C/EBP-Luc containing two copies of a C/EBP binding site, was kindly provided by Peter F. Johnson (NCI-Frederick).

### Adenovirus Transfection

Cells were grown to 90% confluence, infected with Adeno-GFP and Adeno-C/EBPγ, respectively, at 20 MOI. 24 h later, proteins were harvested for the analysis of C/EBPγ expression. In some experiments, the cells were treated with 20 ng/ml IL-1β for the indicated time. The supernatants were collected for ELISA analysis.

### Luciferase Assay

Transient transfections were performed with 8×10^4^ cells plated in 12-well plates by using 0.5 µg of total DNA and 1.5 µl of Fugene®6 Transfection Reagent (Roche, Indianapolis, IN) in 50 µl of Opti-MEM I medium (Invitrogen, Carlsbad, CA). 24 h after transfection, the cells were either incubated with or without 20 ng/ml IL-1β for indicated time. Cell lysates were subjected to luciferase activity analysis by using the Dual-Luciferase Reporter Assay System (Promega, Madison, WI).

### siRNA Transfection

Transient siRNA transfections were performed in 6-well plates by transfecting MLE 12 cells with control siRNA or C/EBPβ/γ siRNA (40 nM) (Santa Cruz, CA) using 5 µl Lipofectamine™ 2000 in 500 µl of Opti-MEM I medium (Invitrogen, Carlsbad, CA). 12 h after siRNA transfection, the cells were treated with or without 20 ng/ml IL-1β for different time points. Supernatants were collected for ELISA.

### ELISA

Both MLE12 cells and primary cultured alveolar type II epithelial cells were stimulated by IL-1β for the indicated time. The supernatants were centrifuged at 3000 rpm for 5 min, and the cell-free supernatants were harvested for IL-6 measurements by using a commercially available ELISA kit (R&D Systems, Minneapolis, MN) according to the manufacturer's protocol.

### RNA Isolation and Detection of mRNA by Quantitative Real-Time PCR (qPCR)

Total RNAs were extracted from cells with Trizol (Invitrogen, Carlsbad, CA) according to the manufacturer's procedure. After isolation, total cellular RNA was incubated with RQ1 RNase-free DNase (Promega, Madison, WI) to remove contaminating DNA. 2 µg of total RNA was submitted to reverse transcription by using the Superscript II RNase H^−^ Reverse Transcriptase (Invitrogen, Carlsbad, CA). The levels of mRNA of C/EBPγ and GAPDH were determined by qPCR. PCR was performed with primers for C/EBPγ: 5′ primer, 5′-GCA AGC AAA GCA AAA AGA GC-3′ and 3′ primer, 5′-GCT TCC AAC CGT TCA TTC TC-3′; GAPDH: 5′ primer, 5′-GCC TCG TCT CAT AGA CAA GAT G-3′ and 3′ primer, 5′-CAG TAG ACT CCA CGA CAT AC-3′. Following reverse transcription, the cDNA (2 µl) was amplified and quantified using a Sequence Detection System (SDS 7300, Applied Biosystems, Foster City, CA) and a PCR universal protocol as follows: AmpliTaq Gold activation at 95°C for 15 s and, annealing/extension at 60°C for 1 min. The fluorescence of the double-stranded products accumulated was monitored in real time. The relative mRNA levels were normalized to levels of GAPDH mRNA in the same sample. The expression of IL-6 by RT-PCR was determined as previously described [28].

### Western Blot Analysis

Both MLE12 and primary cultured AEC II were lysed in cold RIPA buffer. Samples containing 80 µg protein were electrophoresed in a 12% polyacrylamide gel and transferred to a PVDF membrane. Membranes were incubated with rabbit anti-C/EBPγ antibody (provided by Peter F. Johnson) and rabbit anti-GAPDH antibody (Cell Signaling, Danvers, MA), respectively. After 3 washes in TBST, the membranes were incubated with a 1∶5,000 dilution of horseradish peroxidase-conjugated donkey anti-rabbit IgG (GE Healthcare, Piscataway, NJ). The membrane was developed by enhanced chemiluminescence technique according to the manufacturer's protocol (Thermo Fisher Scientific, Rockford, IL).

### Isolation of Alveolar Type II Epithelial Cells

Alveolar type II epithelial cells from C57BL6 mice were isolated and cultured using a method described previously [29]. In all experiments, specific pathogen-free male C57BL/6 mice obtained from Jackson Laboratory (Bar Harbor, ME) were used. All procedures involving mice were approved by the Animal Care and Use Committee of Harvard Medical School. Using biotinylated CD32 and CD45 mAbs, lymphocytes were removed from alveolar type II epithelial cells by streptavidin-magnesphere. The methods achieved high yields (5–7 millions/mouse), high purity (92–95%) and viability (>95%). Alveolar type II epithelial cells were identified by following methods: a) alkaline phosphatase staining [30], b) lamellar body identified by tannic acid staining and by transmission electron microscopy [31], c) immunochemistry for alveolar type II epithelial cells using monoclonal anti-pro-SP-C [31]. Cells were also identified by pan-cytokeratin antibodies (Sigma-Aldrich, St. Louis, MO) [4]. We also used an improved method to maintain the primary cell culture with some modifications [32], [33].

### Transmission Electron Microscopy (TEM)

TEM was used to characterize freshly isolated alveolar epithelial type II cells using modified Karnovsky's fixative [34]. Images were taken and analyzed according to our previous published methods [35], [36], [37].

### Immunostaining

For immunocytochemistry, the cells were fixed in 3.7% paraformaldehyde and non-specific binding was blocked with blocking buffer for 30 minutes [38]. Cells were incubated with primary antibodies at 1/300 dilution in blocking buffer for 1 h and washed three times with wash buffer. After incubation with appropriate fluorophore-conjugated secondary antibodies, the coverslips were mounted on slides with Vectashield mounting medium. The samples were observed on an LSM 510 Meta confocal microscope (Carl Zeiss MicroImaging), and data were processed using the software provided by the manufacturer or Image J software [31].

### Electrophoretic Mobility Shift Assay (EMSA)

Nuclear extracts of MLE12 cells were prepared as follows. Cells were lysed in 15 mM KCl, 10 mM HEPES (pH 7.6), 2 mM MgCl_2_, 0.1 mM EDTA, 1 mM dithiothreitol, 0.1% (v/v) Nonidet P-40, 0.5 mM phenylmethylsulfonyl fluoride, and complete protease inhibitors (Roche, Indianapolis, IN) for 10 min on ice. Nuclei were pelleted by centrifugation at 14,000× g for 20 sec at 4°C. Proteins were extracted from nuclei by incubation at 4°C with vigorous vortexing in buffer C (420 mM NaCl, 20 mM HEPES (pH 7.9), 0.2 mM EDTA, 25% (v/v) glycerol, 1 mM dithiothreitol, 0.5 mM phenylmethylsulfonyl fluoride, and complete protease inhibitors (Roche, Indianapolis, IN). Protein concentrations were determined by Bio-Rad protein assay kit (Thermo Fisher Scientific, Rockford, IL). The EMSA probes were double-stranded oligonucleotides containing a murine IL-6 C/EBP binding site (5′-CTAAACGACGTCACATTGTGCAATCTTAATAAGGTT-3′annealed with 5′-TGGAAACCTTATTAAGATTGCACAATGTGACGTCGT-3′, kindly provided by Richard Schwartz, Michigan State University), or a NF-κB consensus oligonucleotide (AGTTGAGGGGACTTTCCCAGGC, Promega, Madison, WI). C/EBP probes were labeled with α [^32^P] ATP (3,000 Ci/mmol at 10 mCi/ml, GE Healthcare, Piscataway, NJ). NF-κB probes were labeled with γ [^32^P] ATP (3,000 Ci/mmol at 10 mCi/ml, GE Healthcare, Piscataway, NJ). DNA binding reactions were performed at room temperature in a 25 µl reaction mixture containing 6 µl of nuclear extract (1 mg/ml in buffer C) and 5 µl of 5× binding buffer (20% (w/v) Ficoll, 50 mM HEPES pH 7.9, 5 mM EDTA, 5 mM dithiothreitol). The remainder of the reaction mixture contained KCl at a final concentration of 50 mM, Nonidet P-40 at a final concentration of 0.1%, 1 µg of poly (dI-dC), 200 pg of probe, bromphenol blue at a final concentration of 0.06% (w/v), and water to final volume of 25 µl. Samples were electrophoresed through 5.5% polyacrylamide gels in 1× TBE at 190 V for approximately 3.5 h, dried under vacuum, and exposed to X-ray film. For supershifts, nuclear extracts were preincubated with antibodies (1 to 2 µg) for 0.5 h at 4°C prior to the binding reaction. The following antibodies were purchased from Santa Cruz, CA: NF-κB p50, p52, p65, RelB, c-Rel, C/EBPα, C/EBPβ, C/EBPδ, C/EBPε, C/EBPγ, and normal rabbit immunoglobulin G.

### Statistical Analysis

All values were expressed as the mean ± S. E. M. Significance was assigned where *p*<0.05. Data sets were analyzed using Student's *t* test or one-way ANOVA, with individual group means being compared with the Student-Newman-Keuls multiple comparison test.
